# Anxiolytic Effect and Improved Sleep Quality in Individuals Taking *Lippia citriodora* Extract

**DOI:** 10.3390/nu14010218

**Published:** 2022-01-04

**Authors:** Alejandro Martínez-Rodríguez, María Martínez-Olcina, Juan Mora, Pau Navarro, Nuria Caturla, Jonathan Jones

**Affiliations:** 1 Department of Analytical Chemistry, Nutrition and Food Science, University of Alicante, 03690 Alicante, Spain; maria.martinezolcina@ua.es (M.M.-O.); juan.mora@ua.es (J.M.); 2 Alicante Institute of Health and Biomedical Research (ISABIAL), 03010 Alicante, Spain; 3 Monteloeder, S.L., Miguel Servet 16, Nave 17, 03203 Elche, Spain; paunavarro@monteloeder.com (P.N.); nuriacaturla@monteloeder.com (N.C.); jonathanjones@monteloeder.com (J.J.)

**Keywords:** sleep quality, intelligent devices, natural extracts, herbal plants, natural antioxidants, Mediterranean diet, stress, anxiety

## Abstract

The current fast-moving, hectic lifestyle has increased the number of individuals worldwide with difficulties in managing stress, which in turn is also affecting their sleep quality. Therefore, the objective of the current study was to assess a natural plant-based dietary supplement comprised of lemon verbena (*Lippia citriodora*) extract, purified in phenylpropanoids, in alleviating stress and improving quality of sleep. A double-blind, placebo-controlled study was conducted for 8 weeks, followed by a 4-week washout period. Both validated questionnaires and functional tests were performed during the study, whereas questionnaires were used after the washout. As a result, the group taking the lemon verbena extract significantly reduced their perception of stress after 8 weeks, which was corroborated by a significant decrease in cortisol levels. After the washout period, the subjects reported to present even lower stress levels, due to the lasting effect of the ingredient. As for sleep quality, the subjects taking the supplement reported feeling better rested, with a stronger effect observed in women. Sleep tracking using a wearable device revealed that the supplement users improved their times in the deeper stages of sleep, specifically their percentage of time in deep sleep and REM. In conclusion, lemon verbena extract purified in phenylpropanoids is revealed as a natural solution to help individuals to improve their stress and sleep quality.

## 1. Introduction

Anxiety is a normal part of life, and generally is a temporary condition. However, anxiety can occasionally prolong in time, having an impact on everyday activities [1], and giving rise to other conditions, such as depression, drug abuse, cardiovascular disease, and insomnia [2].

There are several synthetic drugs that are commonly used to treat anxiety. These are mainly antidepressants and benzodiazepines [3]. However, their high cost, important side effects and a rising interest in natural solutions have driven researchers to search for botanical-based formulas [4]. To this end, there are a number of plants that have been described to possess potential sedative and anxiolytic effects [5,6,7,8,9,10,11]. Of note is lemon verbena (*Lippia citriodora*), which is native to western South America but is also cultivated in the Mediterranean region and Middle East [12]. The leaves of this plant have been traditionally used to treat various conditions, including fever, digestive discomfort, insomnia and anxiety [13,14]. One of the most abundant polyphenols found in lemon verbena leaves is verbascoside [15], a phenylpropanoid that has been shown to possess antioxidant [16], anti-inflammatory [17] and antimicrobial [18] effects, while also having been shown to promote sleep and alleviate anxiety in mice [19]. In the case of the latter, the mechanism of action that has been described is related to the binding of verbascoside to neural GABA-A receptors, in the same location as benzodiazepines, thereby potentially conferring a similar effect.

Despite its reported traditional use, there are few clinical studies that have assessed the potential anti-anxiolytic and sedative effects of the plant. Therefore, the objective of the current study is to assess the effectiveness of lemon verbena extract, standardized in total phenylpropanoids, on subjects with high levels of perceived stress and low quality of sleep.

## 2. Materials and Methods

### 2.1. Study Design

**Figure 1:**
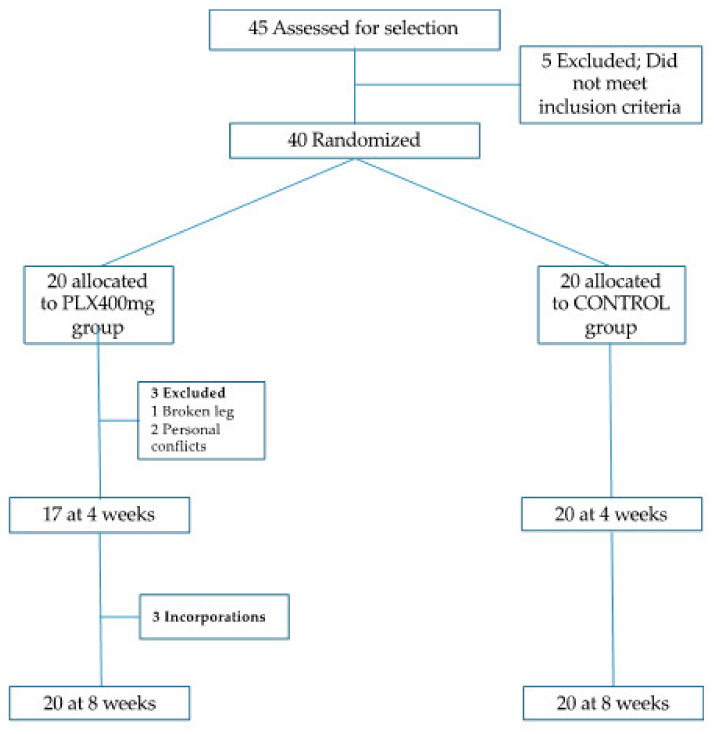
Participant’s flowchart.

This study is a randomized clinical trial in which participants were assigned to one of two groups (Figure 1), to determine the efficacy of a nutraceutical in improving different health parameters. Following published recommendations [20], the subjects were electronically randomized by block design in two arms: an experimental group (lemon verbena extract purified in verbascoside) and a control group (placebo).

### 2.2. Lemon Verbena Extract Characterization

The dietary supplement comprised of a purified extract of lemon verbena leaves (*L. citriodora*), standardized in a minimum of 28% total phenylpropanoids, of which 24% minimum corresponds to verbascoside (commercially known as PLX^®^ or RelaxPLX). This ingredient was provided by Monteloeder SL (Alicante, Spain). The subjects were instructed to take 1 capsule per day, 1–2 h before sleeping. Each capsule contained 400 mg of the lemon verbena extract and 150 mg of excipient (cellulose microcrystalline).

The purification process of the lemon verbena extract was similar to previously published methods [21]. Briefly, dried lemon verbena leaves were mixed with at least 70% ethanol and macerated for 2 h at <80 °C. Then, it was filtered through a silica–cellulose press filter (1 µm) to eliminate insoluble materials, and dried in a vacuum at 60–80 °C. For chromatogram analysis, the extract was dissolved at a concentration of 1 mg/mL.

**Figure 2:**
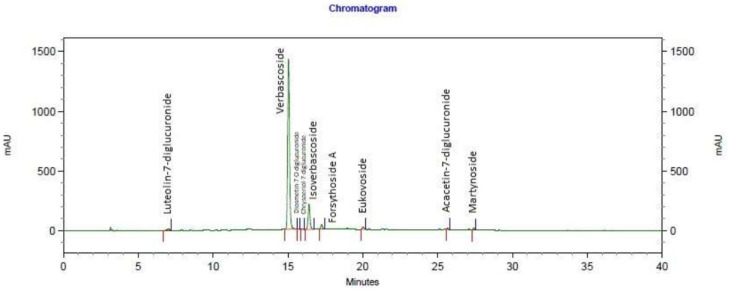
Chromatogram of the lemon verbena extract used in the study, analyzed at 330 nm.

The chromatogram of the lemon verbena extract can be observed in Figure 2 and Table 1. The separation of the compounds from the lemon verbena extract was carried out at room temperature with a gradient elution program at a flow rate of 1 mL/min. The mobile phases consisted in water:acetic acid (2.5%) (A) and acetonitrile (B). The following multi-step linear gradient was applied: 0 min, 5% B; 20 min, 30% B; 30 min, 90% B; 35 min, 5% B; 40 min, 5% B. The initial conditions were held for 10 min. The injection volume in the HPLC system was 20 µL. An HPLC Agilent 1260 Infinity II with an array detector was used. HPLC detection was performed at 330 nm.

### 2.3. Subjects

A total of 40 patients aged 38.7 ± 11.5 years were recruited in several healthcare centers in Elche, Alicante during 2021. All of them were volunteers, the sample consisted of both men and women, and had to meet the following inclusion criteria: (1) over 21 years of age; (2) without any chronic pathology; (3) score on the perceived stress scale (PSS) greater than 15 (moderate levels of stress); and (4) score on the Pittsburgh sleep quality index (PSQI) greater than 5 (poor sleep quality).

### 2.4. Trial Design

**Figure 3:**
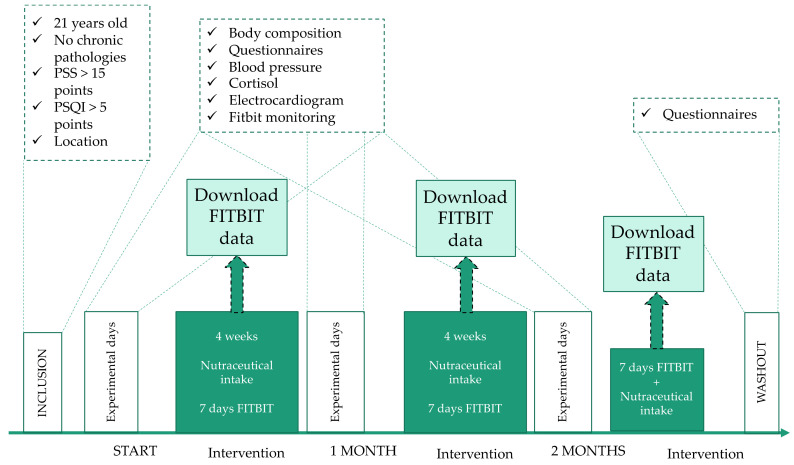
Design and development of the intervention. PSS = perceived stress scale; PSQI = Pittsburgh sleep quality index.

**Figure 4:**
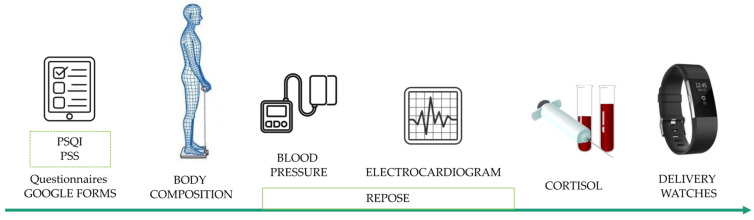
Development of the visits. PSS = perceived stress scale; PSQI = Pittsburgh sleep quality index.

Figure 3 and Figure 4 show the design and development of the research. The intervention lasted 2 months, plus a follow up one month afterwards. Each participant ingested one pill (lemon verbena extract 400 mg or placebo; depending on the group) per day, 1–2 h before bedtime, during the 2 months of investigation. First, participants were assessed to determine whether they met the eligibility criteria, and data was collected on their baseline characteristics (age, weight, height, location). In each of the visits (3 in total) all variables were measured; weight, systolic blood pressure, diastolic blood pressure, height, body composition by electrical impedance (weight, BMI, % fat). Cortisol was also assessed. Each of the volunteers filled out the different questionnaires included in the research (Figure 4) and an electrocardiogram was performed to determine resting heart rate. In addition, the participants wore the Fitbit monitoring bracelet for 7 days at the three time points (baseline—1–2 months). After one month of this last evaluation (three months), the patients retested the questionnaires to study the psychological effect after one month without nutraceutical intake (washout).

### 2.5. Declarations: Ethics Approval, Informed Consent

The present study was conducted in accordance with the norms of the Helsinki declaration. The Human Research Ethics Committee of the University of Alicante (Spain) granted approval to conduct a randomized trial (UA-2021-03-26) and all study participants gave their written consent prior to participation. Moreover, the investigators maintained the confidentiality of all personal data of the participants, coding personal information for this purpose.

### 2.6. Data Collection

#### 2.6.1. Body Composition

In the present investigation, weight and body composition were assessed using the Tanita BC-730F validated bioimpedance scale (Tanita, Amsterdam, The Netherlands). The height of the subjects was measured with a SECA 123 stadiometer (Seca, Hamburg, Germany). From the body mass and height data, BMI (kg/m^2^) was calculated. The aim of making these measurements was to ensure that there were no changes in parameters (e.g., fat mass, weight) that could influence the sleep and stress results.

#### 2.6.2. Sleep Quality Index

The Pittsburgh sleep quality index (PSQI) [22] was used to measure sleep quality. This 24-item questionnaire assesses habitual sleep during the past 30 days. A total of 19 of the questions are self-assessed, with 15 multiple-choice and 4 written, while 5 of the questions refer to the opinion of a “sleeping partner”. According to the PSQI assessment, sleep quality is divided into 7 components: subjective sleep quality, sleep latency, sleep duration, habitual sleep efficiency, sleep disturbances, sleep medication use, and daytime dysfunction. Component scores range from 0 to 3 points. A score of 0 points indicates no difficulty, while a score of 3 indicates severe difficulty. The 7 components are summed to obtain an overall score, which has a range of 0 to 21 points, with a score of 0 points indicating no difficulty, and a score of 21 indicating severe difficulty in all areas studied.

#### 2.6.3. Stress

Perceived stress was analyzed using the perceived stress scale (PSS) questionnaire [23]. The PSS is a valid, reliable, and widely used psychological and psychiatric tool that measures a person’s perception of stress during the previous month. The 10-question version, the PSS-10, was used. Participants rated their emotional and cognitive responses to specific circumstances in their daily life on a 5-point Likert scale, ranging from 0 to 4 (0 = never, 1 = almost never, 2 = sometimes, 3 = quite often, and 4 = very often). The maximum scale score is 40, with higher scores reflecting higher levels of stress and a greater probability of stress interfering with health.

#### 2.6.4. Cortisol Assessment

Cortisol was measured by blood analysis, as an altered stress response and consequent elevated circulating glucocorticoid levels have been found [24] in neuropsychiatric disorders such as depression or anxiety disorders.

#### 2.6.5. Fitbit Sleep Tracking

Sleep-related variables were collected from activity wristbands (Fitbit Charge 2): minutes asleep, times awake, minutes of light sleep, minutes of deep sleep, and minutes of REM. From these data, the percentage of time asleep, light sleep, deep sleep and REM were calculated. The participants wore the activity tracker during the 7 days at the beginning, at 1 month, and at 2 months of the intervention. From these data, the average values were calculated and used for the analysis.

### 2.7. Statistical Analysis

Statistical analysis of the data was performed with the JAMOVI version 1.6.23.0 statistical program (Sydney, Australia). For descriptive statistics (mean ± standard deviation) and inferential analysis, the Shapiro–Wilk test was used to establish the normality distribution. Statistical differences between the different times and groups were tested by ANOVA (general linear model; 3 times × 2 groups)—analysis of variance, with Bonferroni post hoc comparisons. The significance level was set at *p* < 0.05. Effect sizes were calculated with the η2 statistic; an effect of η2 ≥ 0.01 indicates a small effect, ≥0.059 a medium effect, and ≥0.138 a large effect.

## 3. Results

### 3.1. Population Demographics

The study population comprised of 40 individuals, recruited based on their perceived levels of stress and sleep quality. To this end, the PSS score was used to assess anxiety, and the PSQI questionnaire for sleep quality. Participants with a PSS score over 15 and PSQI score over 5 were considered for recruitment. The participants were divided into 2 groups, placebo (*n* = 20; 4 males and 16 females) and experimental (*n* = 20; 10 males and 10 females). Table 2 shows the descriptive data on weight, height, BMI, fat, and visceral fat for each of the groups. No significant changes were observed in body weight/BMI nor fat mass in either of the two groups (Table 3). All were instructed to take the product 1–2 h before going to sleep.

### 3.2. Stress Analysis: Perceived Stress Scale (PSS) and Cortisol Levels

The perceived stress scale (PSS) is a validated questionnaire that allows to measure the amount of stress an individual perceived during the previous month. High values indicate a high level of stress. The results indicated a similar level of stress perceived by both groups during the first month (Table 4 and Table 5). However, during the second month, a significant improvement was detected in the group taking the supplement (−10.7% compared to baseline, *p* < 0.05). During the washout period, the effect of the dietary supplement could still be perceived, with a 20.5% reduction, on average, compared to baseline. A slight tendency to decrease was detected throughout the whole study in the placebo group but did not reach statistical significance. No significant differences were observed in the intergroup analysis.

Cortisol is known as the stress hormone and is a standard method of measuring stress. In the current study, a similar observation was observed as with the PSS questionnaire. Interestingly, decreased cortisol levels were detected in both groups in the first month. However, in the second month, cortisol levels increased in the placebo group to almost baseline levels. In the case of the group consuming the lemon verbena extract, cortisol levels continued decreasing in the second month. Specifically, a 15.6% decrease in cortisol levels were detected in the supplement group after two months of intake compared to baseline, indicating a lower level of stress in this group. The intergroup analysis did not reveal a significant difference, although a tendency to decrease was detected in the second month (*p* = 0.08). Therefore, the group taking the dietary supplement perceived an improved level of stress, coinciding with lower levels of the stress hormone cortisol.

### 3.3. Sleep Analysis: Perceived Sleep Quality Index (PSQI) and Sleep Activity Tracking

Sleep quality was assessed both at the perception level and using a tracking device. The perceived sleep quality index (PSQI) questionnaire was used as the validated test, while Fitbit trackers were provided to the subjects to track their sleep. Regarding the PSQI, a high value indicates the perceived poor quality of sleep. The data from the trackers were collected at the baseline, 1 month, 2 months and after the 1-month washout period. As for the Fitbit trackers, the subjects wore the trackers for 7 consecutive days at the baseline, 1 month and 2 months, and the average values of the parameters assessed were calculated.

Regarding the PSQI questionnaire, results indicated that significant differences were observed in the dietary supplement group starting at the first month, continuing to decrease in the second (−12.2% compared to the baseline, *p* < 0.05) (Table 6 and Table 7). The results after the washout were even more remarked, with a 25.9% decrease compared to the baseline. No significant differences were detected in the placebo group.

When the data was separated based on gender, a more notable effect was observed in the PSQI questionnaire in women (Table 6 and Table 7). In this case, significant differences were observed during the first month, with a 12.7% decrease with respect to baseline values. In the second month, a 22.6% reduction was observed, with similar values obtained during the washout period. No changes were observed in the placebo group in any of the time points analyzed. Significant differences were detected in both vs. the baseline as well as vs. placebo. Thus, the dietary supplement seemed to possess a stronger effect in the females of the study group.

As for the Fitbit data, the following parameters were assessed: %minutes asleep, %minutes awake, number of times awakened, %REM, %deep sleep and %light sleep (Table 8 and Table 9). No significant changes were observed in the placebo group during the whole study, except for a decrease in the number of times awakened during the first month. On the other hand, significant differences were observed in several parameters in the experimental group, regarding the data collected in the second month of the study. Specifically, a significant reduction in the number of times awakened during the night was observed. Additionally, the amount of time while asleep in deep sleep was significantly increased, while REM significantly increased compared to the placebo group. This was observed both in the total population as well as in women only (Table 10 and Table 11). In all cases, the significant differences were observed when the data was compared vs. the baseline, except for %REM where a significant difference vs. the placebo was detected. In the case of women only, %REM was significantly different when comparing with placebo, and %deep sleep vs. baseline values. A tendency to decrease the number of times awakened in women was also detected (*p* = 0.09). As these stages of sleep are associated with mental and physical recovery, it can be assumed that the dietary supplement significantly improved the quality of sleep, and not the quantity. Considering the fact that the individuals slept for an average of 469 and 447 min (approx. 7.5–8 h) in the placebo and experimental groups, respectively, it was not expected to find a significant increase in the minutes asleep.

Therefore, the dietary supplement improved the perceived quality of sleep. Additionally, the number of times the subjects awoke during the night was significantly lower, coinciding with an increased amount of time in the deeper stages of sleep.

## 4. Discussion

In this study, the effect of a dietary supplement comprised of lemon verbena leaf extract was assessed on a population of individuals with moderate levels of stress and poor sleep quality. The toxicity of the lemon verbena extract has been previously studied in rats [25]. That study revealed that the ingredient was safe even at up to at least 2000 mg/kg body weight. As for effective dosages, previous studies have shown that the ingredient was effective for sleep and stress at a daily dosage of 120 mg of verbascoside, albeit with a less concentrated extract. In his study, it was decided to use a more purified extract to see if we could obtain a similar, if not better, effect, while allowing to use a lower dosage that could fit in a single capsule (as opposed to the previous study where the participants had to take two). Several clinical studies have been conducted with the ingredient, with no side effects reported [26,27,28,29,30].

The study population was compared to a placebo group, and analyzed for 8 weeks, followed by a 4-week washout period. A randomized, double-blind placebo-controlled clinical trial was conducted. The results indicate that lemon verbena extracts, purified in the polyphenol verbascoside, may have a significant effect in reducing anxiety when taken continuously for at least 2 months. Specifically, perceived stress decreased after two months, which coincided with lower cortisol levels. Furthermore, the participants taking the lemon verbena extracts reported to perceive improved sleep quality, which was corroborated with an increased amount of time spent in the deeper stages of sleep. Interestingly, a stronger effect on sleep quality perception was observed in women.

Few studies have clinically demonstrated that lemon verbena can improve sleep quality and stress. For example, a clinical study in patients with insomnia reported improved perceived sleep quality after 4 weeks of lemon verbena intake [31]. In another study, lemon verbena extract reduced the number of awakenings during the night, and less reported anxiety after 3 weeks of intake in amateur athletes [27]. However, only validated questionnaires were used in both studies, with no objective analysis included. In the current study, cortisol levels were analyzed in order to assess stress levels and to corroborate with the PSS questionnaire. Similarly, sleep data collected from the Fitbit trackers were used to assess sleep quality. Therefore, the current study provides a more complete, objective assessment of both sleep and stress in individuals with moderate levels of stress and poor sleep quality using lemon verbena extracts.

Fitbit trackers have been used in many other clinical studies, including to assess sleep and anxiety [32,33,34,35]. The advantages of using these trackers are numerous; from being more affordable than medical sleep monitors, their ease of use without requiring technical supervision, their ability to be used for extended period of times, and them being relatively simple to extract data from. Some authors consider that consumer activity trackers may have limited accuracy to measure sleep, especially when compared to gold-standard methods such as polysomnography [36,37]. Despite this, while Fitbit trackers may not be an adequate option to study sleep patterns in disease, for the healthy population it is an affordable, fast, reliable and easy method to collect data.

The mechanisms by which lemon verbena exerts its effects have been partially elucidated. Gamma-aminobutyric acid (GABA) neurotransmitters are known to stimulate sleep and induce relaxation [38]. There are three known GABA receptors, A, B and C, being GABA-A the most relevant for sleep and anxiety. Specifically, there are several methods by which botanical extracts can exert its effect through the GABA system; either by binding to GABA-A receptors to increase its sensitivity to GABA, by increasing GABA synthesis, or increasing GABA release.

In the case of lemon verbena, previous studies have revealed that the major polyphenol verbascoside can bind to the GABA-A receptor in a similar fashion as benzodiazepines, which are regularly used to treat anxiety [39]. In fact, in the referenced study, the sedative effect of lemon verbena extract was enhanced in the presence of diazepam, and its effect decreased when the benzodiazepine receptor antagonist flumazenil was used. Therefore, this mechanism could attribute to the effects observed when consuming the extract.

Several other studies have also elucidated on how lemon verbena and/or verbascoside can induce relaxation, besides GABA-A receptor binding. One study reported that verbascoside exerted its effect via the modulation of cAMP and calcium channels [40]. Furthermore, low doses of verbascoside increased the expression of BDNF, serotonin, noradrenaline and dopamine. Other studies have also analyzed the effect of verbascoside in both animal models and in humans, with plants similar to lemon verbena [41,42].

This research has several strengths; it is a randomized controlled design, sleep quality could be measured both objectively and subjectively, patients could be followed without long lapses of time, CONSORT guidelines were followed and also, the pharmaceutical formulations in the form of a nutraceutical did not cause any patient to not want to participate, as has happened in other investigations with syrups, since sugar also has some biological effects and can affect sleep. However, among the limitations of the present investigation, it should be noted that some authors consider that consumer activity tracking devices, such as Fitbit, may have limited accuracy in measuring sleep, especially when compared to reference methods, such as polysomnography. However, a consumer wearable does not require an external expert, nor sophisticated machinery, which is why it is implemented in research.

## 5. Conclusions

In conclusion, the consumption of a lemon verbena extract purified in verbascoside has been clinically proven to help reduce anxiety and improve quality of sleep. Previous studies suggest that the mechanism of action is at least partially based on the binding of the polyphenol to GABA-A receptors in the same binding sites as benzodiazepines. Therefore, these results suggest the plausibility of using lemon verbena extracts as a natural alternative to sedative drugs, that possess many side effects and can cause dependency.

## Figures and Tables

**Table 1:** HPLC analysis compounds.

Name	Retention Time
Luteolin-7-diglucuronide	7.013
Verbascoside	15.040
Diosmetin-7-O-diglucuronide	15.720
Chrysoeriol-7-diglucuronide	15.987
Isoverbascoside	16.413
Forsythoside A	17.253
Eukovoside	20.027
Acacetin-7-diglucuronide	25.667
Martynoside	27.427

**Table 2:** Basic anthropometric and demographic characteristics of the sample.

Variable	Placebo Group (*n* = 20)	Experimental Group (*n* = 20)
	Mean ± SD	Mean ± SD
Age (years)	43.4 ± 13.9	34.0 ± 6.28
Height (cm)	164 ± 8.08	173 ± 9.07
Weight (kg)	63.2 ± 10.7	80.8 ± 10.7
BMI (kg/m^2^)	23.5 ± 3.0	26.7 ± 4.5
Body fat (%)	23.8 ± 8.17	25.7 ± 8.81
Visceral fat	4.47 ± 2.60	5.11 ± 2.84

*n* = sample number; SD = standard deviation; cm = centimeters; kg = kilograms; % = percentage.

**Table 3:** Body composition values table.

Variable	Group	Baseline	Month 2
		Mean ± SD	Mean ± SD
Weight	Placebo	63.2 ± 10.9	63.3 ± 11.0
	Experimental	80.8 ± 16.2	80.0 ± 15.8
BMI	Placebo	23.5 ± 3.1	23.5 ± 3.0
	Experimental	26.7 ± 4.5	26.5 ± 4.3
Fat Mass	Placebo	24.5 ± 8.2	23.5 ± 9.1
	Experimental	25.4 ± 10.3	24.9 ± 10.0

SD = standard deviation.

**Table 4:** Chart depicting values; anxiety tests.

Variable	Group	Baseline	Month 1	Month 2	Washout
		Mean ± SD	Mean ± SD	Mean ± SD	Mean ± SD
PSS Score	Placebo	22.3 ± 7.1	21.5 ± 8.3	20.7 ± 7.6	20.2 ± 6.6
	Experimental	20.8 ± 7.1	20.3 ± 6.6	18.7 ± 5.7*	17.1 ± 7.6 *
Cortisol	Placebo	16.6 ± 5.6	14.3 ± 4.8 ***	15.7 ± 4.9	NA
	Experimental	15.1 ± 6.0	13.7 ± 5.7 **	12.6 ± 6.0***	NA

SD = standard deviation; PSS = perceived stress scale; NA = no data analysis. * *p* < 0.05, ** *p* < 0.01, *** *p* < 0.001 vs. baseline. ANOVA test were performed.

**Table 5:** Results of anxiety tests. Change vs. baseline = percent change from baseline.

Variable	Group	Month 1 vs. Baseline	Month 2 vs. Baseline	Washout vs. Baseline
		Mean ± SD	Mean ± SD	Mean ± SD
PSS Score	Placebo	0.96 ± 0.24	0.93 ± 0.27	0.91 ± 0.41
	Experimental	0.98 ± 0.24	0.90 ± 0.27 *	0.82 ± 0.25 *
Cortisol	Placebo	0.87 ± 0.11 ***	0.94 ± 0.31	NA
	Experimental	0.91 ± 0.08 **	0.83 ± 0.31 ***	NA

SD = standard deviation; PSS = perceived stress scale; NA = no data analysis. * *p* < 0.05, ** *p* < 0.01, *** *p* < 0.001 vs. baseline. ANOVA test were performed.

**Table 6:** Chart depicting values.

Variable	Group	Baseline	Month 1	Month 2	Washout
		Mean ± SD	Mean ± SD	Mean ± SD	Mean ± SD
PSQI Score (Total)	Placebo	8.5 ± 2.9	7.8 ± 2.3	7.7 ± 2.6	7.3 ± 2.8
	Experimental	6.7 ± 2.0	6.2 ± 2.2 *	5.9 ± 2.6 *	5.0 ± 2.4 **
PSQI Score Males	Placebo	5.5 ± 0.6	5.38 ± 2.6	6.4 ± 4.6	4.9 ± 2.8
	Experimental	8.7 ± 3.2	8.7 ± 1.9	8.0 ± 2.5	7.8 ± 3.1*
PSQI Score Females	Placebo	8.3 ± 2.7	8.1 ± 2.4	8.3 ± 2.4	7.9 ± 2.5
	Experimental	7.3 ± 2.6	6.5 ± 2.4 *	5.7 ±2.5 *^†^	5.6 ± 2.7 **^†^

SD = standard deviation; PSQI = Pittsburgh sleep quality index. * *p* < 0.05, ** *p* < 0.01 vs. baseline. ^†^ *p* < 0.05 vs. placebo. ANOVA test were performed.

**Table 7:** Results of PSQI questionnaire. Change vs. baseline = percent change from baseline.

Variable	Group	Month 1 vs. Baseline	Month 2 vs. Baseline	Washout vs. Baseline
		Mean ± SD	Mean ± SD	Mean ± SD
PSQI Score (Total)	Placebo	0.92 ± 0.24	0.91 ± 0.42	0.86 ± 0.48
	Experimental	0.93 ± 0.21 *	0.88 ± 0.34 *	0.75± 0.25 **
PSQI Score Males	Placebo	0.98 ± 0.18	1.18 ± 0.65	0.86 ± 0.36
	Experimental	0.99 ± 0.33	0.92 ± 0.65	0.89 ± 0.36
PSQI Score Females	Placebo	0.98 ± 0.23	1.00 ± 0.41	0.95 ± 0.48
	Experimental	0.88 ± 0.21 *	0.78 ± 0.28*^†^	0.76 ± 0.22 **^†^

SD = standard deviation; PSQI = Pittsburgh sleep quality index. * *p* < 0.05, ** *p* < 0.01 vs. baseline. ^†^ *p* < 0.05 vs. placebo. ANOVA test were performed.

**Table 8:** Chart depicting Fitbit values in total population.

Variable	Group	Baseline	Month 1	Month 2
		Mean ± SD	Mean ± SD	Mean ± SD
% Minutes Asleep	Placebo	87.7 ± 1.7	87.6 ± 2.2	88.6 ± 3.0
	Experimental	84.9 ± 1.0	87.8 ± 1.8	88.4 ± 2.3
% Minutes Awake	Placebo	12.3 ± 1.7	12.4 ± 2.2	11.3 ± 2.9
	Experimental	12.1 ± 2.1	12.2 ± 1.7	11.6 ± 2.3
Number of Times Awakened	Placebo	24.2 ± 4.9	20.9 ± 5.2 *	20.6 ± 6.7
	Experimental	28.8 ± 8.7	29.9 ± 7.8	22.9 ± 7.2 *
% REM	Placebo	20.2 ± 4.7	23.2 ± 8.1	19.0 ± 5.2
	Experimental	21.4 ± 5.0	21.2 ± 5.3	23.0 ± 5.1 ^†^
% Light Sleep	Placebo	65.3 ± 6.8	70.4 ± 12.2	65.1 ± 10.1
	Experimental	64.6 ± 9.9	62.9 ± 6.5	61.3 ± 7.1
% Deep Sleep	Placebo	15.2 ± 5.4	16.9 ± 7.1	16.8 ± 3.6
	Experimental	16.7 ± 3.4	16.6 ± 3.4	18.6 ± 3.7 *

SD = standard deviation; % = percentage. * *p* < 0.05 vs. baseline, ^†^ *p* < 0.05 vs. placebo.

**Table 9:** Results of Fitbit data. Change vs. baseline = percent change from baseline.

Variable	Group	Month 1 vs. Baseline	Month 2 vs. Baseline
		Mean ± SD	Mean ± SD
% Minutes Asleep	Placebo	0.99 ± 0.13	1.01 ± 0.10
	Experimental	1.03 ± 0.10	1.04 ± 0.07
% Minutes Awake	Placebo	1.01 ± 0.13	0.92 ± 0.21
	Experimental	1.01 ± 0.13	0.96 ± 0.16
Number of Times Awakened	Placebo	0.86 ± 0.19 *	0.85 ± 0.28
	Experimental	1.04 ± 0.36	0.79 ± 0.29 *
% REM	Placebo	1.05 ± 0.09	0.94 ± 0.18
	Experimental	1.08 ± 0.29	1.07 ± 0.25 ^†^
% Light Sleep	Placebo	1.08 ± 0.06	0.99 ± 0.13
	Experimental	0.97 ± 0.15	0.95 ± 0.13
% Deep Sleep	Placebo	1.11 ± 0.06	1.11 ± 0.28
	Experimental	0.99 ± 0.13	1.11 ± 0.27 *

Results of Fitbit data. Change vs. baseline = percent change from baseline. SD = standard deviation; % = percentage. * *p* < 0.05 vs. baseline, ^†^ *p* < 0.05 vs. placebo. ANOVA test were performed.

**Table 10:** Chart depicting Fitbit values in women population.

Variable	Group	Baseline	Month 1	Month 2
		Mean ± SD	Mean ± SD	Mean ± SD
% Minutes Asleep	Placebo	87.7 ± 1.8	87.3 ± 2.2	88.0 ± 2.3
	Experimental	88.1 ± 2.4	87.6 ± 1.9	89.2 ± 1.7
% Minutes Awake	Placebo	12.3 ± 1.8	12.6 ± 2.2	11.8 ± 2.1
	Experimental	11.8 ± 2.2	12.3 ± 1.9	10.7 ± 1.8
Number of Times Awakened	Placebo	23.5 ± 5.3	20.6 ± 5.5	21.0 ± 5.4
	Experimental	25.6 ± 9.4	28.1 ± 7.6	19.1 ± 7.6
% REM	Placebo	20.6 ± 5.2	22.9 ± 8.3	19.3 ± 5.8
	Experimental	21.5 ± 6.0	21.1 ± 6.5	26.9 ± 2.7 ^†^
% Light Sleep	Placebo	66.3 ± 7.1	70.9 ± 13.0	66.4 ± 11.1
	Experimental	67.7 ± 12.5	65.1 ± 8.1	58.8 ± 8.6
% Deep Sleep	Placebo	14.1 ± 5.4	16.2 ± 7.3	16.0 ± 3.6
	Experimental	15.8 ± 3.9	15.2 ± 3.5	19.1 ± 4.5 *

SD = standard deviation; % = percentage. * *p* < 0.05 vs. baseline, ^†^ *p* < 0.05 vs. placebo. ANOVA test were performed.

**Table 11:** Fitbit values in women population. change vs. baseline = percent change from baseline.

Variable	Group	Month 1 vs. Baseline	Month 2 vs. Baseline
		Mean ± SD	Mean ± SD
% Minutes Asleep	Placebo	0.99 ± 0.14	1.00 ± 0.02
	Experimental	0.99 ± 0.07	1.01 ± 0.01
% Minutes Awake	Placebo	1.02 ± 0.15	0.96 ± 0.13
	Experimental	1.04 ± 0.12	0.91 ± 0.05
Number of Times Awakened	Placebo	0.88 ± 0.49	0.89 ± 0.24
	Experimental	1.10 ± 0.19	0.75 ± 0.41
% REM	Placebo	1.11 ± 0.21	0.94 ± 0.21
	Experimental	0.98 ± 0.07	1.25 ± 0.24 ^†^
% Light Sleep	Placebo	1.07 ± 0.14	1.00 ± 0.14
	Experimental	0.96 ± 0.08	0.87 ± 0.17
% Deep Sleep	Placebo	1.15 ± 0.42	1.13 ± 0.28
	Experimental	0.96 ± 0.08	1.22 ± 0.29 *

SD = standard deviation; % = percentage. * *p* < 0.05 vs. baseline, ^†^ *p* < 0.05 vs. placebo. ANOVA test were performed.

## Data Availability

The data presented in this study is available on request from the corresponding author. The data are not publicly available due to is personal health information.
